# Neonate Pain Management: What do Nurses Really Know?

**DOI:** 10.5539/gjhs.v6n5p284

**Published:** 2014-07-15

**Authors:** Fariba Asadi-Noghabi, Mina Tavassoli-Farahi, Hadi Yousefi, Tahereh Sadeghi

**Affiliations:** 1 Student Research Committee, Shiraz University of Medical Sciences, Shiraz, Iran; 2 Health Information Management Research Center, Hormozgan University of Medical Sciences, Bandar Abbas, Iran; 3 School of Nursing & Midwifery, Hormozgan University of Medical Sciences, Bandar Abbas, Iran; 4 Department of Neonatal Intensive Care, Faculty of Nursing and Midwifery, Tehran University of Medical Sciences, Tehran, Iran

**Keywords:** pain, neonate, pain management

## Abstract

**Purpose:**

The purpose of this study was to determine knowledge, attitude, and performance vis-à-vis pain management in neonates by nurses working in neonatal units in Bandar Abbas University hospitals.

**Method:**

This descriptive and analytical study was executed from March-August 2011 in the neonatal units and NICU in Bandar Abbas educational hospitals. A total of 50 nurses and nurse assistants working in the neonatal units participated in the study. The data collection tool was a structured questionnaire investigating knowledge (28 items), attitude (20 items) and practices (5 items). Data was analyzed using descriptive statistical tests (Frequency, Mean and Standard deviation tables) and inferential statistic (T-test, Variance analysis).

**Results:**

The knowledge scores of participants had a mean value of 13.51 (48.2%) out of 28. The mean score of attitude was 54.22 out of 60 and the mean score for the nurses’ level of practices was found to be 4.22 out of 10. There was a significant relationship between nurses’ knowledge scores and the level of education, i.e. nurses with more education had more knowledge.

**Conclusion:**

Results showed that the nurses had poor performance regarding the assessment, measurement, and relief of pain. However, they showed positive attitudes towards pain control in neonates.

## 1. Introduction

According to the International Association for the Study of Pain, pain is defined as “an unpleasant sensory and emotional experience associated with actual or potential tissue damage” (Williams & Manias, 2008). Casey in Sadeghi et al. (2013) pain is known as the fifth vital sign, and health professionals should monitor and manage it when caring for patients (Sadeghi, Mohammadi, Shamshiri, Bagherzadeh, & Hossinkhani, 2013). The anatomic and physiological origin for the perception of painful stimuli exists even in extremely preterm neonates. Infants who are treated in the neonatal intensive care unit often feel pain, usually for protracted periods of time. Newborn infants have an increased sensitivity to pain and are more reactive to pain than older children and adults, and are susceptible to long-term pain-associated effects (Bartocci, Bergqvist, Lagercrantz, & Anand, 2006; Slater et al., 2006). Studies have shown that inadequate management of pain in preterm infants may cause perpetual alteration in brain processing and maladaptive behavior later in life (Anand, 2000). Pain may also have certain damaging impact on the subsequent ability of an infant to learn and remember new information. Prolonged stress due to pain also leads to permanent decrease of hippocampal dendrites. Recurrent pain and/or stress affect this apoptotic action more seriously (Buskila et al., 2003). Pain in newborns is a common phenomenon and all infants are regularly exposed to pain early in their lives. In a study by Simons carried out on 151 neonates, an average of 14 ± 4 painful interventions/day was recorded for the first 14 days of life (Simons et al., 2003). Non-pharmacological pain intervention is a prophylactic and complementary approach to alleviate pain. A number of non-pharmacological therapies have shown to be effective in the management of mild to moderate pain in neonates. These therapies include the non-nutritive sucking (NNS) both with and without sucrose, breast milk, breastfeeding, swaddling or facilitated tucking, kangaroo care, music therapy, and multi-sensorial stimulation (Golianu, Krane, Seybold, Almgren, & Anand, 2007; Kashaninia, Sajedi, Rahgozar, & Noghabi, 2008; Sajedi, Kashaninia, Rahgozar, & Noghabi, 2007). Most medical and nursing procedures still cause pain in children. Nurses need to know useful measures to control or relieve children’s pain (Sadeghi et al., 2013; Mcnair et al., 2013). The integral responsibility of health care professionals caring for children is to remove pain and suffering when possible. Yet, studies have demonstrated that neonates are often under-treated for their pain (Simpkin, Robertson, Barber, & Young, 2009). Recent findings suggest that the number of neonates exposed to pain during hospitalization is exceedingly high due to inadequate pain relieving methods (Stevens et al., 2003). Unsubstantiated beliefs, insufficient knowledge among caregivers, and inadequate application of knowledge contribute to the lack of adequate management. Personal values and beliefs of health care professionals about the meaning and value of pain in the development of a child (*e.g*., the belief that pain builds character) and the treatment of pain may interfere with the optimal recognition and treatment of pain for all children (Vallano, Malouf, Payrulet, & Baños, 2006). Recent literature related to the issue of pain management suggests that there are limitations in the area of nursing knowledge associated with clinical pain management techniques (Oldenmenger, Sillevis Smitt, van Dooren, Stoter, & van der Rijt, 2009; Smeltzer, Bare, Hinkle, & Cheever, 2009; Thomas, 1997; Wilson, 2007). To improve the recognition and treatment of pain in sick children, the health care professionals need to expand their knowledge, use appropriate assessment tools and techniques, anticipate painful experiences and intervene accordingly, use a multimodal approach in managing pain, use a multidisciplinary approach when possible, involve families, and advocate for the use of effective pain management in children (Layman Young, Horton, & Davidhizar, 2006; Ware, Bruckenthal, Davis, & O’Conner-Von, 2011; Foster, 2013). Many studies have documented nurses’ lack of knowledge regarding the physiology of pain, pain assessment, pharmacological and non-pharmacological interventions for pain management (Bernardi, Catania, & Tridello, 2007; Gunnarsdottir, Donovan, Serlin, Voge, & Ward, 2002; Johnson, Kassner, Houser, & Kutner, 2005; Mrozek & Werner, 2001; Ware et al., 2011; Zaza & Baine, 2002). To improve this aspect of care, it is desirable to assess the knowledge, attitude and practices of nurses. However, in Iran, there has been little assessment of the knowledge, attitudes, and practices of nurses concerning pain management in children. Therefore, this survey was undertaken to assess knowledge, attitudes, and practices among nurses involved in managing pain in neonates.

## 2. Material Studied

### 2.1 Setting and Sampling

This was a descriptive-analytical study carried out at neonatal and NICU wards of two hospitals (Children’s Medical Center) affiliated to Hormozgan University of Medical Sciences, Bandar Abbas, Iran, from March-August 2011. These two hospitals are supposed to offer high quality and specialized therapeutic services to neonates, infants and children throughout region being a teaching hospital. Inclusion criteria for participantion in the study included all postgraduate and graduate nurses and working in neonatal and NICU (Neonatal Intensive Care Unit) wards. Sampling for this study was a census. All nurses who worked in the neonatal and NICU were invited to participate in this research.50 questionnaires were distributed, but 40 usable questionnaires were returned.

### 2.2 Instruments

A survey questionnaire for nurses’ knowledge, attitudes, and pain management practices in neonates was used to collect the data. The questionnaire was developed by the researcher based on PNKAS (Nurses Knowledge and Attitudes Survey regarding pain) questionnaire. It consisted of demographic characteristics of participants (6 items); a checklist of nurses’ pain management practices (5 items) with total score ranging from 0-10 and examined the modality and type of interventions for pain relief; nurses’ knowledge in pain management (28 true/false questions) scoring from 0–28; and nurses’ attitudes in pain management (20 items) based on a 3-point rating scale ranging from 1 (disagree) to 3 (agree). The total score ranged from 20–60. To eliminate the effect of pain management practices on nurses’ knowledge and attitudes, the checklist concerning the nurses’ practice in pain management was simultaneously completed by an expert nurse (observer) and the researcher and later, the questionnaire regarding the knowledge and attitude was distributed among nurses eligible to enter the study. The coefficient between the two observers was determined by calculating Kappa coefficient (0.78). Content validity of the instruments was established through revision by a number of faculty members skilled in pain management. The suggestions made were incorporated into the questionnaire. Internal reliability for the questionnaire was 93.

### 2.3 Ethical Considerations

Permission was obtained from the head nurses of neonatal and NICU wards of teaching hospitals affiliated to the Hormozgan University of Medical Sciences, Bandar Abbas. Nurses were assured that their name would remain completely anonymous. The participants were informed of the purpose of the study and how their confidentiality was assured. Ethical clearance was obtained from the Hormozgan University of Medical Sciences’ Ethics Committee.

### 2.4 Data Analysis

Data analysis was performed using SPSS 16.0. Descriptive statistics (frequency, percentage, mean, standard deviation) were used to examine the normality of the data and to describe the sample. Independent t-tests and one-way analysis of variance were used to examine the differences in knowledge scores with categorical demographic variables. A p value less than .05 was considered as significant, statistically.

## 3. Results

### 3.1 Sociodemographic Characteristics

Of the 50 distributed questionnaires, 40 usable questionnaires were returned, giving a response rate of 80%. All Subjects were within the age group of 24-51 years, with an average age of 32 years. The demographic data also indicated that the majority (85.5%) of respondents were female aged below 35, 75% held a graduate degree in nursing, and more than one-thirds (35.5%) were officially employed. Majority of respondents (90%) were married and nearly two-thirds (72.5%) had children.

### 3.2 Knowledge in Pain Management

The mean value for the nurses’ knowledge in pain management was 13.51 (48.2%) out of 28 with scores ranging from a minimum of 3 to a maximum of 19. Additional item analysis of the subjects’ knowledge was performed to determine the areas where they might have/lack correct knowledge.

Tables 1 and 2 present the items with the highest and lowest frequencies and the percentage of correct answers, respectively. As shown in table 1, the five items with the highest frequencies associated with the correct answers were: item 20 (90%), followed by items 4 (80%), 2 (77.5%), 18 (77.5%), and 16 (70%).

**Table 1:** The highest frequency and percentage of items for nurses’ knowledge with correct answers (N=40)

Items no.	Items	Correct responses	n	%
20	Direct skin contact of mother and neonate is a good way to relieve pain in neonates	T	36	90.0
4	Children who need to frequently undergo painful procedures, need the maximum treatment for pain control during the first procedure to minimize the anxiety for the next procedures	T	32	80.0
2	Children sleep in spite of severe pain	T	31	77.5
18	Research shows that lapping neonates during a painful procedure does not usually have an effect on the child’s pain intensity	F	31	77.5
16	Research shows that music has no effects on neonate’s pain intensity, but has a sedative effect on older children	F	30	75.0
28	When performing a painful procedure, breast feeding will relieve pain in neonates	T	30	75.0
15	Non-pharmacologic methods of pain relief have no applications for neonates	F	28	70.0
10	After an initial does of opioid analgesic is given, subsequent doses should be adjusted in accordance with the individual patient’s response	T	28	70.0
1	Neonates who can be distracted from pain usually do not have severe pain	F	27	67.5
11	Neonates respond to drug treatment less than adults	F	26	65.0
13	Opioids use in children may lead to physical dependence which is different from addiction	T	26	65.0
9	Neonates less than 6 months cannot tolerate opioids for pain relief	F	25	62.5
17	Oral administration of glucose to neonates before painful procedures can reduce pain	F	25	62.5

*Abbreviations: T, true; F, false*.

The items with the lowest frequencies in correct answers were 24 (0%), 27 (0%), 26 (7.5%), 6 (12.5%), 23 (15%), 12 (17.5%), and 8 (22.5%), respectively (Table 2). The highest percentage of correct answers belonged to the non-pharmacological pain management and the lowest to the pharmacological interventions and its complications in neonates. No statistical difference existed in knowledge scores associated with the respondents’ education level, marital status, number of children, and the type of employment.

**Table 2:** The lowest frequency and percentage of items for nurses’ knowledge with correct answers (n=40)

Items no.	Items	Correct Responses	n	%
**24**	Paracetamol is the most commonly used analgesic in neonates	T	0	0.0
**27**	The likelihood of drug addiction is less than 40 percent through use of opioid analgesics for pain relief	T	0	0.0
**26**	Ibuprofen is appropriate for relief of mild pain in neonates	F	3	7.5
**6**	The World Health Organization pain ladder suggests using a single analgesic rather than combining classes of drugs (e.g. combining an opioid with a non-steroidal agent)	F	5	12.5
**23**	Morphine is not used for neonate pain relief due to the risk of respiratory depression	F	6	15.0
**12**	Anti-anxiety drugs, Sedatives, and Barbiturates are suitable for pain relief during painful procedures	T	7	17.5
**8**	Research shows that Promethazine (Phenergan) is a reliable potentiator of opioid analgesics	T	9	22.5
**7**	The usual duration of action of meperidine is 4 to 5 hours	T	11	27.5
**5**	Respiratory depression rarely occurs in neonates who have been receiving opioids over a period of months	T	13	32.5
**25**	Nonsteroidal, anti-inflammatory agents are used to relieve mild to moderate pain	T	13	32.5
**19**	Swaddling the neonate during painful procedures causes limitation of motions which results in increased pain	F	15	37.5
**3**	Aspirin and other nonsteroidal anti-inflammatory agents are NOT effective analgesics for painful bone metastases	F	16	40.0
**14**	Opioids should not be administered for neonate pain relief, due to the high risk of psychological dependence.	F	17	42.5
**21**	Intramuscular injection is the recommended route of administration of opioids for neonates with brief, severe pain of sudden onset as trauma or postoperative pain	F	24	60.0
**22**	Analgesics for post-operative pain should initially be given around the clock on a fixed schedule	T	24	60.0

*Abbreviations: T, true; F, false*.

### 3.3 Attitudes Towards Pain

Statistical analysis revealed a mean score of 54.22 out of 60 for nurses’ attitude ranging from 46 to 59. The majority of participants (36; 90%) showed positive attitudes towards the neonates’ pain assessment and measurement (scored above 50).

Table 3 exhibits the nurses’ answers to the items on attitudes. As shown, 90% of the respondents believed that the neonates have the right to be appropriately assessed and managed for their pain (item 4). Most individuals (67.5%) emphasized on the importance of the role they have in assessing and measuring pain in neonates (item 7). Most respondents highlighted the significance of assessment tools for effective pain measurement and adoption of appropriate pain relief methods which facilitate the process of pain alleviation in neonates (items 15, 17, 18). There was a significant difference between the respondents’ knowledge and the attitude scores viz., nurses with higher level of knowledge in neonates’ pain relief showed more positive attitudes (p < 0.05).

**Table 3:** Participants’ responses to items on the nurses’ attitudes survey regarding pain assessment and relief

Sl. no.	Items	Agree		Not sure		Disagree	

		n	%	n	%	n	%
**1**	Neonates and children experience pain equal to that experienced by adults	38	95.0	0	0.0	2	5.0
**2**	Parents should not be present during painful procedures	9	22.5	2	5.0	29	72.5
**3**	Pain management and pain relief are of priority in neonates treatment	32	80.0	6	15.0	2	5.0
**4**	Neonates have the right to appropriate assessment and management of their pain	36	90.0	3	7.5	1	2.5
**5**	The most accurate judge of the intensity of the neonate’s pain is the her/his primary nurse	37	92.5	3	7.5	0	0.0
**6**	Full treatment of pain is a humanitarian issue	21	52.5	4	10.0	15	37.5
**7**	To better assess neonate pain, the nurse can discuss with her/his parents	27	67.5	9	22.5	4	10.0
**8**	Assessment and control of neonate pain lead to improved his/her parents satisfaction	35	87.5	3	7.5	2	5.0
**9**	Failure to assess and manage the neonate’s pain affects his body and mind in the long term	34	85	3	7.5	4	10.0
**10**	The nurse’s physical and mental fatigue can affect neonate pain relief	39	97.5	0	0.0	1	2.5
**11**	Like other vital signs, pain scores should be documented	36	90	1	2.5	3	7.5
**12**	To ensure patient’s comfort and pain relief is one of the most important tasks of nurses	32	80	4	10.0	4	10.0
**13**	Communicating with and educating neonate’s parents play an effective role in relieving pain	38	95.0	1	2.5	1	2.5
**14**	Available tools for measurement of pain are the best for determining pain severity in neonate	38	95.0	1	2.5	1	2.5
**15**	When the necessary procedures have been done for the patient, the persistence of pain does not cause problems	23	27.5	8	20.0	9	22.5
**16**	Using pain assessment tools for determining neonate’s pain lead to an appropriate method of pain relief	30	75.0	5	12.5	5	12.5
**17**	Measurement and control of neonate’s pain can affect the healing process and reduces the hospital stay	28	70.0	10	25.0	2	5.0
**18**	Evaluation and measurement of neonate’s pain should be considered as one of the vital signs when examining the neonate	39	97.5	0	0.0	1	2.5
**19**	Comparable stimuli in different people produce the same intensity of pain	38	95.0	2	5.0	0	0.0
**20**	Measurement and control of pain in neonate leads to improved quality of neonate’s life	39	97.5	1	2.5	0	0.0

### 3.4 Performance in Pain Management

Out of a possible 10 points, the average score for the nurses’ practice in pain management was 4.22 which varied from 0 to 6. Tables 4 and 5 represent the nurses’ use of pharmacological and non-pharmacological methods of pain relief. Almost all nurses (100%) used pharmacological and non-pharmacological interventions from time to time nevertheless; they never took any pain relief measure during painful procedures such as blood sampling, intramuscular injection (IM), and venipuncture procedures (Tables 4 and 5).

**Table 4:** Use of pharmacologic methods for pain relief in neonates (N = 40)

Frequency	n	%
**Never**	0	0.0
**Sometimes**	40	100.0
**Always**	0	0.0

**Table 5:** Use of non-pharmacologic methods for pain relief in neonates (N = 40)

Frequency	n	%
**Never**	0	0.0
**Sometimes**	40	100.0
**Always**	0	0.0

## 4. Discussion

Nurses have a crucial role in the assessment, management, and alleviation of patients’ pain. In fact, they are the main observers of patients’ pain and suffering, acting as liaisons between nurses and patients. The nurses participated in this study showed low level of practices regarding the pain relief in neonates. In most cases, they failed to adopt any measure to lessen pain in neonates during painful procedures including blood sampling, intramuscular injection (IM), and procedures, among others. Lack of knowledge among nurses, as well as the negative attitudes towards pain experience of neonates have been described to be the main barriers in pain assessment and alleviation (Elcigil, Maltepe, Esrefgil, & Mutafoglu, 2011; Ely, 2001; Igier, Mullet, & Sorum, 2007; Layman Young et al., 2006; Potts & Mandleco, 2011). Nurses in our study showed knowledge deficits over pain management in neonates. The mean value obtained for their knowledge in pain management was 13.51 (48.2%) points out of 28. These findings are somehow in accordance with a study by LeLande performed in southern Florida, where he found that the Oncology Certified Nurses scored significantly higher (mean = 71%) than did the Non-Certified Oncology Nurses (mean = 62%). The nurses also knew little about pharmacology in particular when it was required to administer an analgesic, ordered “as needed” to maintain a steady state of analgesia (LaLande, 2010). Subhashni carried out a study entitled “Knowledge, attitude and practices among nurses regarding pain” in New Delhi, India. He found that nearly two-thirds of the respondents (62.3%) felt that the non-pharmacological measures are better to control pain rather than the drugs; of these the most common method reported was the act of distraction such as use of music. One-third (32.4%) of nurses believed that a child complaining of pain despite increasing the amount of analgesic was only due to development of physiological dependence (Subhashini, Vatsa, & Lodha, 2009). In a study by Yildirim on knowledge and attitudes of Turkish oncology nurses about cancer pain management, the average correct response rate was 35.41% (Yildirim, Cicek, & Uyar, 2008). A comparative study of the knowledge on pain and pain management between the nurses from UK, South Africa and Sweden, revealed different levels of knowledge and positive attitudes to pain management among nurses, with Swedish nurses at higher levels of knowledge and a more positive attitude to pain management than the nurses from UK or South Africa. A high level of knowledge correlated with more positive attitude to pain management (Enskär et al., 2007). Al-Shaer examined a total of 129 registered nurses (RNs) over the knowledge and attitudes regarding the pain assessment and intervention. Out of a possible 32 points, the average knowledge score was 25.9. Scores ranged from a minimum of 20 to a maximum of 31. In her study, like this study, the most frequently incorrect responses belonged to the pharmacological methods for pain relief. About 76% of nurses missed the item “Aspirin 650 mg PO is approximately equal in analgesic effect to meperidine 50 mg PO”, nevertheless they scored higher on non-pharmacological strategies for pain management. Nearly 60% agreed that “the non-drug interventions (e.g., heat, music, imagery, etc.) are very effective for mild-moderate levels of pain, but rarely helpful for more severe pain” (Al-Shaer, Hill, & Anderson, 2011). Mathew and others performed a study to assess the knowledge, attitude and practice of pediatric critical care nurses towards pain and the results unveiled that all respondents believed that neonates feel pain; however 50% of nurses felt that neonates perceive pain less than adults (Mathew, Mathew, & Singhi, 2011). Parvizi and others in their study on nurses’ problems in applying non-pharmacological pain management for children showed that only 3.3% of nurses knew about the non-pharmacological pain management methods. Two major causes of problems in most nurses (82.2%) were due to inadequate training during their education and missing the continued education courses during their nursing work (Parvizi, ELHANI, & Aghebati, 2008). *V*arvani-Farahani evaluated the effects of establishing a nursing commission for pain management to empower nurses during pain assessment process. Results indicated that the nurses’ competence in assessing the pain in neonates was very low (7.7%) (Varvani Faraahani, Elhani, & Mohammadi, 2009)

In this present study, the mean score of nurses’ attitudes in assessing and measuring pain in neonates was 54.22 out of a maximum of 60 points ranging from 46 to 59. A vast majority (36; 90%) of nurses showed positive attitudes towards assessment and measurement of pain in neonates (scored above 50). The majority of nurses believed that neonates have the right to appropriate assessment and management of their pain which have a positive effect on their healing process. Enkser et al. (2007) found that the Swedish nurses not only have higher levels of knowledge, but also a more positive attitude towards pain management. Layman and others in their study on nurses’ attitudes and beliefs over pain assessment and management found that most nurses believed that pain assessment instruments could improve the nurse-patient relationship resulting in better alleviation of pain. They failed to find any significant relationship between the amount of experience and the nurses’ attitudes towards pain, a finding that’s in line with this present study. However, a significant relationship between the amount of trainingand the nurses’ attitudes to pain was identified (Layman et al., 2006). Lui investigated the level of knowledge and attitudes regarding pain management among nurses working in medical units in Hong Kong results showed that 23.8% of respondents never used any pain assessment tools. Only 20.3% of nurses claimed that they often used such tools for pain measurement. The mean score of knowledge in pain management was 9.49 out of 26, indicating of a deficit in knowledge. These nurses possessed better attitudes to pain compared to the previous study. They enumerated staff shortage and heavy workload as the most important barriers to effective pain measurement and management (Lui, So, & Fong, 2008)

Negative attitudes of caregivers are among the most important factors impeding effective pain management in neonates, hence, assessing their attitudes towards pain measurement and management will pave the way for initiating appropriate strategies for altering negative attitudes in assessment, recognition, and treatment of pain in neonates. Fortunately, most participants in this study had positive attitudes to pain management in children. However, positive attitude alone does not necessarily mean adequate pain management practices. Factors such as sufficient knowledge also contribute to the effective control of pain. In the present study, a low level of knowledge about pain management was observed, implying an inadequacy in nursing practice in the assessment and management of pain in neonates. However, a significant difference between the nurses’ educational levels and the scores of knowledge was observed. With regard to this finding, there are conflicting results reported in different studies. De Rond et al. (2000) and Clarke et al. (1996) reported significant differences between the nurses’ knowledge and amount of experience. On the contrary, Wilson (2007) did not find such differences, a finding consistent with the results of our study.

## 5. Conclusion

The findings of this study suggest that the participants need in-depth education regarding pharmacologic and non-pharmacologic strategies for pain alleviation in neonates. To address this problem effectively, training programs concerning knowledge of pain in neonates; adverse effects and measurement of pain; and drug/non-drug relieving interventions are recommended. Training programs not only enhance the knowledge and attitudes of nursing personnel, but they might also lead to optimal pain management in neonates. Further investigations would also help to identify and resolve the problems hindering effective pain relief in neonates.
